# Mulberry Leaf (*Morus alba* L.) Extracts and Its Chlorogenic Acid Isomer Component Improve Glucolipotoxicity-Induced Hepatic Lipid Accumulation via Downregulating miR-34a and Decreased Inflammation

**DOI:** 10.3390/nu14224808

**Published:** 2022-11-14

**Authors:** Tsung-Yuan Yang, Meng-Hsun Yu, Yi-Liang Wu, Ching-Chun Hong, Chin-Shuh Chen, Kuei-Chuan Chan, Chau-Jong Wang

**Affiliations:** 1 Department of Internal Medicine, Chung-Shan Medical University Hospital, No. 110, Section 1, Jianguo N. Road, Taichung 402, Taiwan; 2 School of Medicine, Institute of Medicine, Chung-Shan Medical University, No. 110, Section 1, Jianguo N. Road, Taichung 402, Taiwan; 3 Department of Health Industry Technology Management, Chung Shan Medical University, No. 110, Section 1, Jianguo N. Road, Taichung 402, Taiwan; 4 Department of Nutrition, Chung Shan Medical University, No. 110, Section 1, Jianguo N. Road, Taichung 402, Taiwan; 5 Division of Cardiovascular Surgery, Surgical Department, Chung Shan Medical University Hospital, No. 110, Section 1, Jianguo N. Road, Taichung 402, Taiwan; 6 Department of Surgery, School of Medicine, Chung-Shan Medical University, No. 110, Section 1, Jianguo N. Road, Taichung 402, Taiwan; 7 Department of Food Science and Biotechnology, National Chung Hsing University, Taichung 402, Taiwan; 8 Department of Medical Research, Chung Shan Medical University Hospital, No. 110, Section 1, Jianguo N. Road, Taichung 402, Taiwan

**Keywords:** mulberry leaf extract, neochlorogenic acid, nonalcoholic fatty liver disease, hepatic lipid accumulation and inflammation, miR-34a

## Abstract

Mulberry leaf (*Morus alba* L.) is used as a traditional medicine and potential health food to treat various metabolic diseases, such as hypertension, diabetes, and hyperlipidemia. However, we sought the mechanisms by which functional components of mulberry leaves mediate diabetic steatohepatitis. We applied an in vitro model of HepG2 cells induced by glucolipotoxicity and evaluated the effects of MLE and its major components nCGA, Crp, and CGA. The results showed that MLE and nCGA reduced liver fat accumulation by inhibiting SREBP-1/FASN, SREBP-2/HMG-CoAR, and activating PPARα/CPT-1. Additionally, MLE and nCGA decreased inflammatory responses associated with NF-κB, TNF-α, and IL-6 to alleviate steatohepatitis. Furthermore, we showed that MLE and nCGA exerted anti-glucolipotoxicity effects by downregulating miR-34a, thus activating SIRT1/AMPK signaling, and subsequently suppressing hepatic lipid accumulation.

## 1. Introduction

Diabetes mellitus is mainly caused by insulin resistance, and patients with diabetes often also have other metabolic abnormalities such as obesity, hypertension, and hyperlipidemia. Excessive body fat accumulation can cause NAFLD or diabetic steatohepatitis, which is a common complication of diabetes. If left untreated, diabetic steatohepatitis can lead to liver fibrosis or cirrhosis, and even induce hepatocellular carcinoma [1]. Therefore, the prevention and treatment of diabetic steatohepatitis has become an important health issue worldwide. Chronic glucolipotoxicity and insulin resistance can promote the progression of NAFLD to diabetic steatohepatitis, and persistent hyperglycemia is a key phenomenon observed in the course of subclinical inflammation and diabetic steatohepatitis [2]. In addition, hepatic inflammation can increase the influx of free fatty acids to the liver, inducing intrahepatic triglycerides and subsequently lipogenesis and hepatocellular injury via lipid peroxidation and mitochondrial dysfunction [3]. Furthermore, lipotoxicity-induced ballooning can lead to a vicious circle of cell stress and apoptosis in steatohepatitis.

MicroRNAs (miR) are small non-coding RNAs that affect insulin resistance, adipogenesis, and lipid metabolism [4]. They also play an essential role in the regulation of disorders associated with lipid metabolism, including metabolic syndrome and atherosclerosis [5]. Adenovirus-mediated miR-34a expression in the liver has been associated with an increase in NAFLD, whereas inhibition of miR-34a expression has been demonstrated to have the opposite effect [6,7]. However, the role of hepatocyte miR-34a in the progression of NAFLD to steatohepatitis or its reversal remains to be elucidated. The potential target genes of these miRs include sterol regulatory element-binding protein 1 (SREBP-1) and SREBP-2, which are involved in the synthesis of hepatic lipids [8]. SREBP-1 enhances the transcription of genes required for fatty acids and has been linked with fatty acid synthesis; SREBP-2 modulates the transcription of genes involved in cholesterol metabolism [9]. Rspo1 and Rspo3 have been shown to reverse oleic acid (OA)-induced cholesterol synthesis, promote the phosphorylation of adenosine monophosphate-activated protein kinase (AMPK), and attenuate the expression of SREBP-2 [10]. Sirtuin 1 (SIRT1) has been shown to play a critical role in the ability of resveratrol to stimulate AMPK and improve mitochondrial function [11]. In addition, several nuclear receptors, including proliferator-activated receptor alpha (PPARα), AMPK and SREBP, have been shown to play key roles in the regulation of lipid homeostasis during the pathogenesis of NAFLD [12]. A mice model found that antrodan could alleviate high-fat and high-fructose diet-induced NAFLD via the AMPK/SIRT1/SREBP/PPARα pathway [13]. AMPK is an enzyme which is essential for the regulation of either anabolism or catabolism by directly phosphorylating proteins or modulating gene transcription in multiple pathways, such as fatty acid oxidation, synthesis, and lipolysis of lipids [14]. Therefore, the SIRT1/AMPK pathway is a promising target in the prevention of steatohepatitis.

The expressions of fatty acid synthase (FASN) and hydroxymethylglutaryl-CoA reductase (HMG-CoAR), enzymes essential for the regulation of lipid homeostasis, are considered to be markers of cholesterol synthesis [12]. Conversely, the expressions of carnitine palmitoyltransferase-1 (CPT-1) and PPARα play key roles in the upregulation of fatty acid oxidation. A previous animal model showed that high-glucose-fed rats had increased liver damage caused by high levels of nuclear factor Kappa-B (NF-κB), tumor necrosis factor alpha (TNF-α) and interleukin 6 (IL-6) [15]. During NAFLD, NF-κB is responsible for the production of pro-inflammatory cytokines such as IL-6 and TNF-α [16]. Decreased NF-κB activity in liver macrophages has also been associated with decreased liver inflammation [17].

Mulberry leaf (*Morus alba* L.) is a genus of flowering plants belonging to the Moraceae family, which is widely cultivated in China, Japan, and Korea [18], and it contains numerous chemical phytochemicals, including Deoxynojirimycin (DNJ), phenolics, anthocyanins, flavonoids, and polyphenolic amides [19]. In addition, we isolated and identified the polyphenolic compounds contained in mulberry leaf extract (MLE), which mainly involved three chlorogenic acid (CGA) isomers, namely neochlorogenic acid (nCGA), cryptochlorogenic acid (Crp), and CGA. However, the mechanisms by which MLE and its main components ameliorate diabetic steatohepatitis have not been clarified. In addition, the anti-inflammatory mechanisms of CGA isomers on lipid metabolism are also unknown. Here, we established an in vitro model of glucolipotoxicity (OA + high glucose; OH)-induced lipid accumulation and inflammation in HepG2 cells. Our hypothesis was that CGA isomers may alleviate hepatic lipid accumulation through several mechanisms, including attenuation of inflammatory responses via inhibition of NF-κB and TNF-α-related signaling and downregulation of miR-34a, leading to activation of the SIRT1/AMPK pathway and subsequently modulation of lipid β-oxidation enzymes and the lipogenesis-associated transcription factors. Targeting these mechanisms may search a novel approach for the treatment of diabetic steatohepatitis.

## 2. Results

### 2.1. Effect of Viability on HepG2 Cells Treated with MLE and CGA Isomer

**Figure 1:**
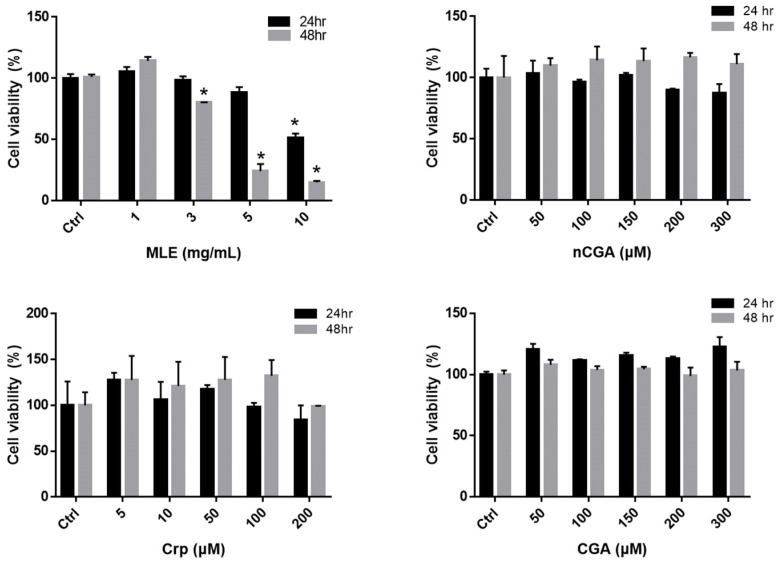
HepG2 cells were treated with different concentrations of MLE (1.0, 3.0, 5.0, and 10.0 mg/mL) and nCGA, Crp and CGA (0, 50, 100, 150, 200 and 300 μM) for 24 and 48 h. Determine the cell viability of HepG2 cells by the MTT assay. Values are the mean ± SD of three replicates per treatment. * *p* < 0.05 vs. the control group.

The viability of HepG2 cells exposed to MLE and CGA was evaluated using the MTT assay. As indicated in Figure 1, HepG2 cells exhibited no cytotoxicity after treatment with different concentrations of MLE (1.0–10.0 mg/mL) and nCGA (0–300 μM) for 24 or 48 h. Furthermore, treatment with different concentrations of Crp (0–300 μM) and CGA (0–300 μM) for 24 or 48 h was not cytotoxic to HepG2 cells. Consequently, these results demonstrate that CGA isomer treatments had no significant effects on HepG2 cell viability and serve as the basis for subsequent experiments.

### 2.2. Effect of Oleic Acid and High Glucose-Induced Lipid Accumulation

**Figure 2:**
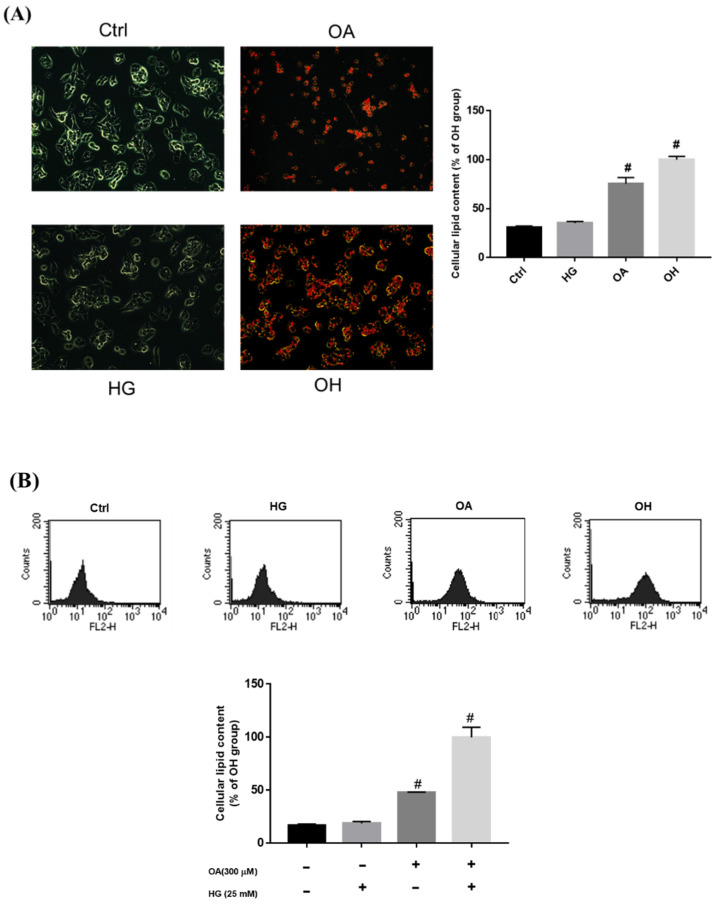
Treatment with OA and HG−induced lipid accumulation in HepG2 cells. HepG2 cells were treated with 25 mM HG, OA, and OH for 24 h. Intracellular lipid accumulation was measured by (**A**) oil red O staining and (**B**) Nile red staining. Values are mean ± SD for three replicates per treatment. ^#^ *p* < 0.05 vs. the control group.

A previous study revealed that OA could accelerate lipid accumulation [20]. Therefore, we established a model of glucolipotoxicity using 25 mM high glucose (HG), 300 μM OA, and OH to stimulate the production of lipid accumulation in the HepG2 cell for 24 h, respectively. (Figure 2A). Oil red O staining demonstrated that OA and OH treatment induced lipid accumulation in the HepG2 cells, and the lipid content was significantly higher in the OH groups. The HepG2 cells were stained with Nile red and measured using flow cytometry, which also showed that the lipid content was significantly higher in the OH groups (Figure 2B).

### 2.3. Inhibitory Effect of MLE and CGA Isomers on Lipid Accumulation

HepG2 cells were treated with OH and various concentrations of MLE (1, 3, or 5 mg/mL) or CGA isomers at 37 °C for 24 h. Intracellular lipid accumulation was measured using oil red O staining, which showed that the lipid accumulation in the HepG2 cells was inhibited when the concentration of MLE was ≥3.0 mg/mL or nCGA, Crp, and CGA were ≥50 μM, ≥100 μM, and ≥200 μM, respectively. Quantization was determined using colorimetric analysis, and lipid accumulation was shown to be induced by OH.

**Figure 3:**
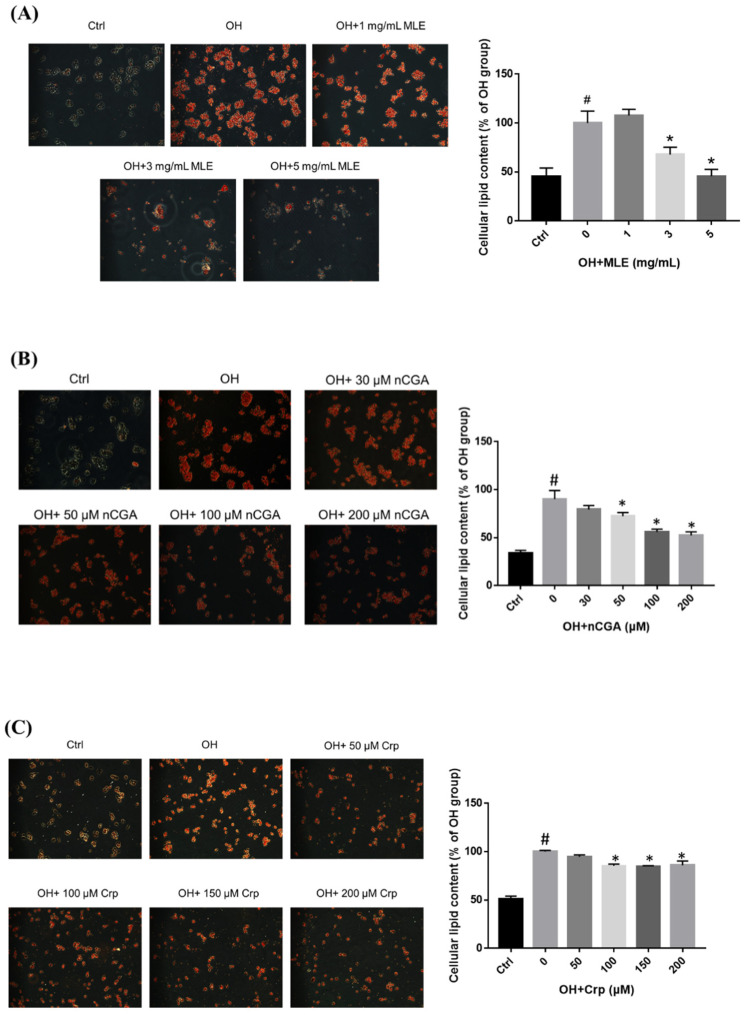

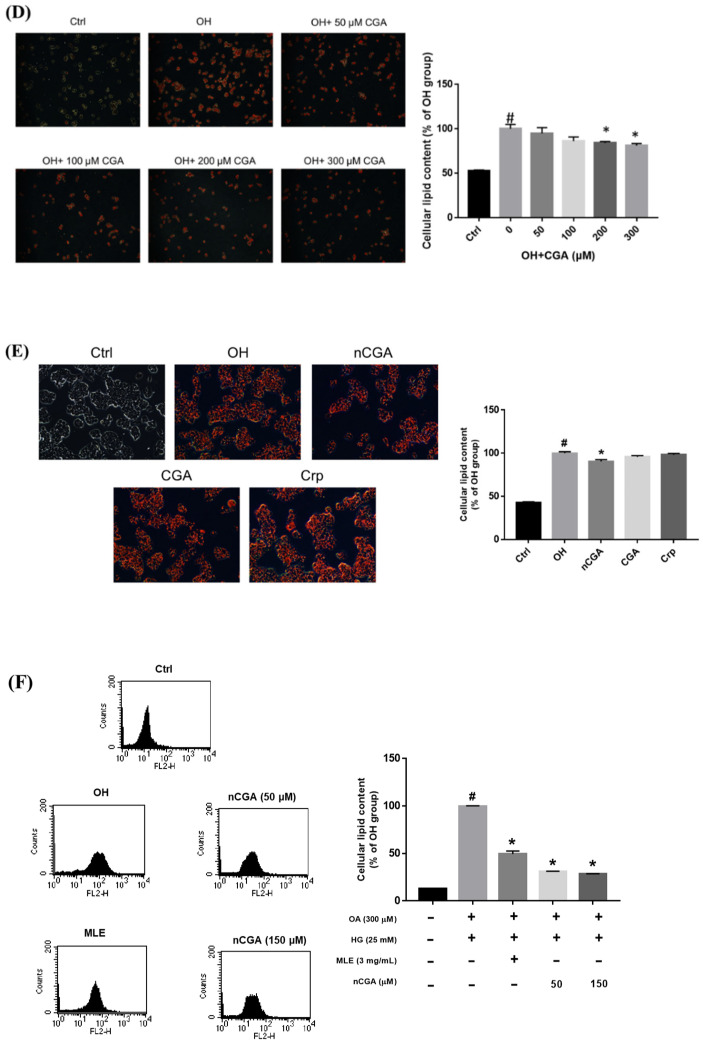
Effect of MLE and CGA isomers on lipid accumulation in OH−treated HepG2 cells. HepG2 cells were treated with 300 μM oleic acid + 25 mM high glucose (OH) and various concentrations of (**A**) MLE (**B**) nCGA (**C**) Crp and (**D**) CGA at 37 °C for 24 h. Intracellular lipid accumulation was measured by oil red O staining. (**E**) The different alleviation effects of these CGA isomers on lipid accumulation in the HepG2 cells; nCGA had the most significant alleviation effect when compared with the other two CGA isomers. (**F**) The lipid accumulation in HepG2 cells was inhibited as determined by Nile red staining. Values are the mean ± SD from three replicates per treatment. ^#^ *p* < 0.05 vs. the control group. * *p* < 0.05 vs. the OH group.

We then evaluated the different alleviation effects of these three CGA isomers at the same concentration of 50 μM. OH-cultured HepG2 cells were treated with 50 μM of the CGA isomers at 37 °C for 24 h. Intracellular lipid accumulation was measured using oil red O staining (Figure 3E), which showed that the lipid accumulation in the HepG2 cells was inhibited only by 50 μM nCGA, but not by 50 μM Crp or 50 μM CGA. In other words, nCGA had the strongest alleviation effect when compared with the other two CGA isomers, suggesting that nCGA may be the major functional component of MLE. Other OH-cultured HepG2 cells were treated with 3 mg/mL MLE and 50/150 μM nCGA at 37 °C for 24 h (Figure 3F). The lipid accumulation in the HepG2 cells was also significantly inhibited when the MLE concentration was 3.0 mg/mL and nCGA concentration was 50 or 150 μM.

### 2.4. MLE and nCGA Activated the AMPK Signaling Pathway and β-Oxidative Signal Pathway in OH-Cultured HepG2 Cells

**Figure 4:**
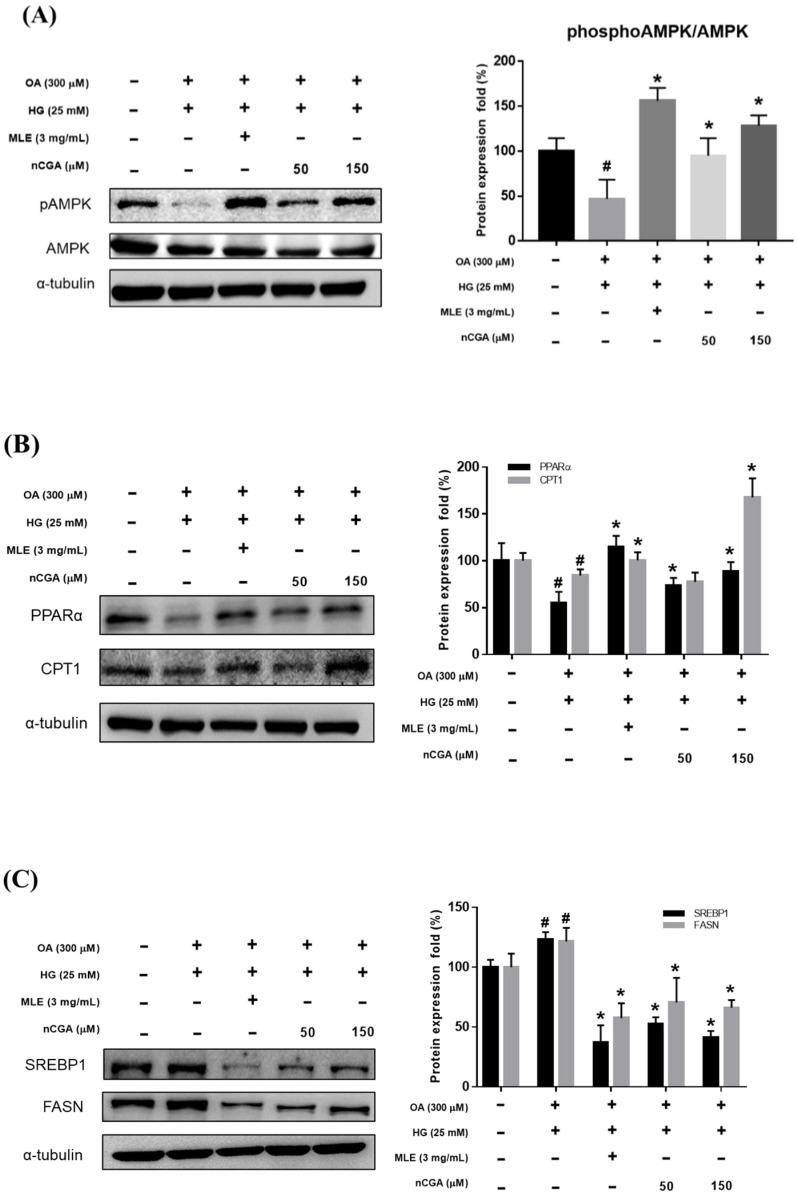

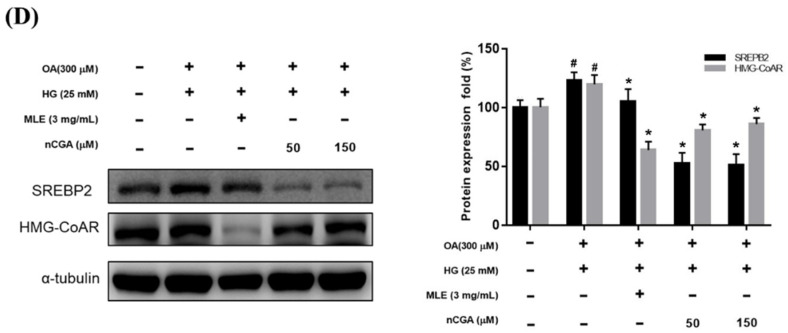
Effects of MLE and nCGA on multiple signaling pathways in OH−induced HepG2 cells. HepG2 cells were co-treated with OH, MLE (3 mg/mL), and nCGA (50 or 150 μM) at 37 °C for 24 h. Protein extracts were measured by Western Immunoblotting; α-tubulin was used as an internal control. MLE and nCGA activated (**A**) pAMPK/AMPK and (**B**) PPARα/CPT-1 signaling pathways in OH-induced HepG2 cells, but reduced (**C**) SREBP-1/FASN and (**D**) SREBP-2/HMG-CoAR protein expressions. Values are the mean ± SD of three independent experiments. ^#^ *p* < 0.05 vs. the control group. * *p* < 0.05 vs. the OH group.

AMPK has been reported to be a mediator of lipogenesis and to play a critical role in the pathogenesis of steatohepatitis. As shown in Figure 4A, OH treatment significantly reduced the expression of pAMPK/AMPK compared with the controls, while co-administration with MLE (3 mg/mL) and nCGA (50 or 150 μM) reversed the effects.

CPT-1 is a rate-limiting enzyme in the mitochondrial response of fatty acid β-oxidation. The results of Figure 4B show that the excessive accumulation of lipids in HepG2 cells induced by OH, the expression of CPT-1 was significantly reduced compared with the control group. After treatment with MLE (3 mg/mL) and nCGA (150 μM), the CPT-1 protein showed a significant increasing trend, indicating that MLE and nCGA can promote fatty acid β-oxidation.

### 2.5. MLE and nCGA Reduced Lipogenesis-Related Protein Expression in OH-Cultured HepG2 Cells

To determine whether MLE and nCGA reduce the expression of hepatic lipid accumulation, we investigated the expression of lipogenic proteins such as SREBP, FASN, and HMG-CoAR. HepG2 cells were co-treated with OH, MLE (3 mg/mL), and nCGA (50 or 150 μM) at 37 °C for 24 h. Protein extracts from the HepG2 cells were measured by Western blotting to detect SREBP-1, FASN, SREBP-2 and HMG-CoAR; α-tubulin was used as a loading control. OH significantly promoted the expressions of SREBP-1 and FASN (Figure 4C). Furthermore, MLE and nCGA significantly suppressed the OH-induced expressions of SREBP-2 and HMG-CoAR (Figure 4D), which are involved in cholesterol synthesis.

### 2.6. Effects of MLE and nCGA on Fatty Acid Oxidation-Related and Inflammatory Protein Expressions in OH-Induced HepG2 Cells

**Figure 5:**
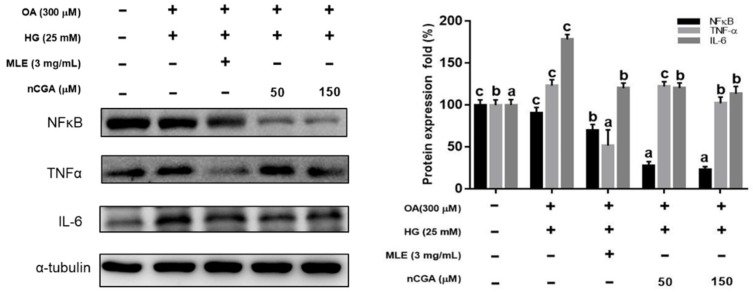
MLE and nCGA reduced inflammatory protein expressions in OH−induced HepG2 cells. HepG2 cells were co-treated with OH, MLE (3 mg/mL), and nCGA (50 or 150 μM) at 37 °C for 24 h. Protein extracts from the HepG2 cells were measured by Western blotting to detect TNF-α, NF-κB, and IL-6; α-tubulin was used as a loading control. Values are the mean ± SD of three independent experiments. Data were represented using one-way ANOVA, followed by Duncan’s new multiple range test. Quantitative bars not sharing a common letter are significantly different (*p* < 0.05) from each other.

Previous reports have indicated that the upregulation of PPARα and increased fatty acid oxidation may protect against lipid accumulation and steatohepatitis [21,22]. Therefore, we evaluated the effects of MLE and nCGA on the expressions of PPARα and CPT-1, which are involved in fatty acid oxidation. The results shown in Figure 4B, OH significantly inhibited the expressions of PPARα and CPT-1. Furthermore, co-treatment of OH with MLE (3 mg/mL) and nCGA (50 or 150 μM) increased the expressions of PPARα and CPT-1. TNF-α is *critically* involved in the *pathophysiology of hepatocellular* inflammation and steatohepatitis. Our results showed that OH significantly promoted the expressions of IL-6 and TNF-α (Figure 5). In addition, co-treatment of OH with MLE and nCGA reduced the expressions of NF-κB, TNF-α and IL-6.

### 2.7. MiR-34a Inhibitor Activated SIRT1, p-AMPK/AMPK, and PPARα Signaling Pathways in OH-Induced HepG2 Cells

**Figure 6:**
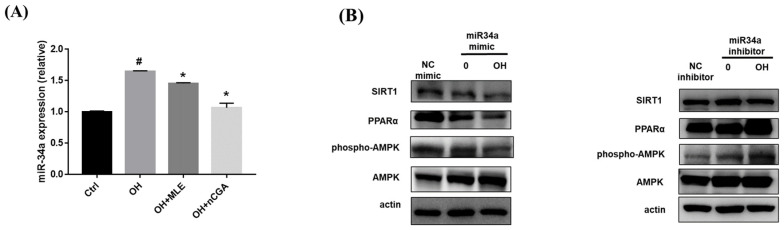
MLE and nCGA reduced miR-34a expression in OH−induced HepG2 cells. (**A**) HepG2 cells were co-treated with OH, MLE (3 mg/mL), and nCGA (50 μM) at 37 °C for 24 h. The level of miR-34a was analyzed using Real-Time PCR. (**B**) Transfection with miR-34a mimics or inhibitors in the HepG2 cells were measured by Western blotting to detect SIRT1, PPAR-α, and p-AMPK/AMPK. Values are the mean ± SD of three independent experiments. Data were represented using the Student’s *t*-test. ^#^ *p* < 0.05 vs. control group. * *p* < 0.05 vs. the OH group.

MiR-34a is thought to play a key role in NAFLD and diabetic steatohepatitis [23]. Mechanistically, inhibiting miR-34a could block the development of steatohepatitis by decreasing lipid accumulation, and apoptosis. As shown in Figure 6, OH treatment significantly increased the expression of miR-34a compared with the controls, whereas co-administration with MLE (3 mg/mL) and nCGA (50 μM) reversed the effects. Furthermore, treatment with an inhibitor of miR-34a increased the expressions of SIRT1, p-AMPK/AMPK, and PPARα. These results showed the effects of an miR-34a inhibitor on the expressions of SIRT1, p-AMPK/AMPK, and PPARα, which are involved in the lipogenesis and β-oxidative signal pathway.

**Figure 7:**
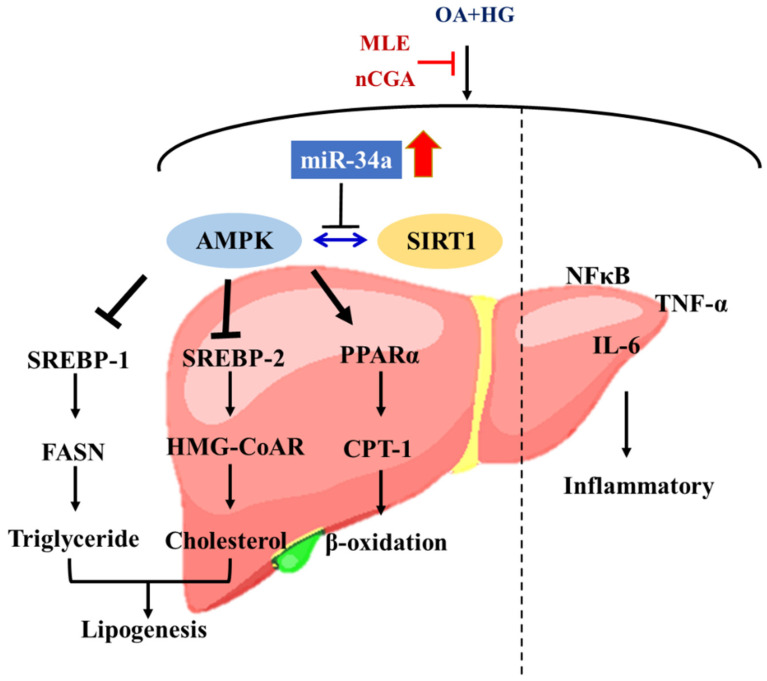
The MLE and nCGA decreased inflammation by inhibiting NF-κB signaling, and regulating the SIRT1-AMPK pathway via miR-34a reduced lipogenesis.

In summary, our results demonstrated that MLE and nCGA could inactivate miR-34a, leading to up-regulation of the SIRT1/AMPK signal, which modulates the expressions of lipid β-oxidation enzymes and lipogenesis-associated transcription factors such as SREBP-1, SREBP-2, FASN, and HMG-CoAR, or inhibition of inflammatory processes, leads to remission of hepatic lipid accumulation. MLE and nCGA also reduced the expressions of NF-κB, TNF-α and IL-6, resulting in an anti-inflammation of glucolipotoxicity (Figure 7).

## 3. Discussion

The number of patients with NAFLD and diabetic steatohepatitis is rapidly increasing worldwide, consistent with the increased prevalence of metabolic syndrome. The metabolic syndrome is characterized by a group of conditions including hypertension, diabetes, hyperlipidemia, and obesity; the major risk factors for NAFLD. Diabetes promotes the progression of NAFLD to steatohepatitis, with histological findings including Mallory-Denk bodies, lobular inflammation, hepatocyte ballooning degeneration, and pericellular fibrosis [24]. Furthermore, steatohepatitis can advance to a more serious form such as cirrhosis, or potentially to liver-related morbidity or mortality due to hepatic failure or hepatocellular carcinoma [25]. Therefore, approaches that suppress lipogenesis and inflammatory responses would be expected to prevent the development of steatohepatitis. With recent advances in diabetic steatohepatitis-specific therapies, it is increasingly important for clinical physicians to identify patients with diabetic steatohepatitis and initiate an integrated multidisciplinary program of treatment to achieve the best possible outcomes.

Increasing evidence has demonstrating that miRs regulate pathways involving lipid homeostasis, oxidative stress and inflammatory responses in the pathophysiology of steatohepatitis [22,26]. MiR-34a is thought to play a key role in NAFLD and diabetic steatohepatitis. A recent study found that hepatocyte miR-34a was a pivotal regulator in the progression from NAFLD to steatohepatitis [7]. Mechanistically, inhibiting miR-34a could block the development of steatohepatitis by decreasing lipid accumulation, and apoptosis. Therefore, we investigated the mechanisms by which miR-34a influences steatohepatitis through multiple pathways. The downregulation of miR-34a led to the activation of SIRT1, AMPK, PPARα and various downstream genes [21], moreover treatment with an miR-34a inhibitor contributed to the attenuation of lipogenesis and improved steatohepatitis. SIRT1 is another regulator of hepatic lipid homeostasis, and it plays an important role in hepatic steatosis. Guo et al. reported that metformin alleviated steatohepatitis in a SIRT1-dependent manner [27]. Another study showed that a PPARα agonist could improve insulin sensitivity and lipid metabolism, potentially inducing the resolution of steatohepatitis without worsening fibrosis [28]. Other data have demonstrated that the loss of *Arid1a* enhances NAFLD by downregulating PPARα, thereby blocking fatty acid oxidation and leading to lipogenesis and insulin resistance [29,30]. These findings are consistent with the present study, in which we demonstrated that MLE and nCGA had the effect of silencing miR-34a directly and targeting the transcription factors SIRT1, AMPK, and PPARα. Downregulation of miR-34a may be a therapeutic strategy against glucolipotoxicity-induced steatohepatitis by regulating its targets PPARα and SIRT1.

The CPT-1 pathway plays a crucial role in β-oxidation, and the attenuation of mitochondrial fatty acid oxidation due to the suppression of PPARα and CPT-1 is an important event in the pathogenesis of steatohepatitis [31]. In the present study, we found that the inhibition of miR-34a activated SIRT1/AMPK and the expression of CPT-1, leading to the β-oxidation of mitochondria. These observations may at least partially explain the mechanism by which increased β-oxidation after the inhibition of miR-34a alleviates glucolipotoxicity-induced steatohepatitis. Our results also showed that AMPK was a mediator of elevated CPT-1 levels after MLE and nCGA treatment.

In addition, the SREBP family have been shown to play a key role in lipogenesis, including cholesterol and triglyceride synthesis. Persistent SREBP activation has been shown to result in the development of steatohepatitis and atherosclerosis [32]. Kim et al. reported that endoplasmic reticulum stress drives lipogenesis and steatohepatitis via caspase-2 activation, and that this induces the expression of SREBP-1/2 [33]. SREBP-1 is the main transcriptional regulator of FASN, and corresponding protein levels of FASN have been reported in human and animal models of NAFLD. The expression of FASN in liver cells may be considered a compensatory adaptation in the early stages of NAFLD or steatohepatitis [32,34]. Ren et al. reported that suppressing the expressions of SREBP-2 and HMG-CoAR could attenuate NAFLD in diabetic rats [35]. Multiple signaling pathways can promote the activation of SREBP-2 to control the HMG-CoAR pathway and regulate cholesterol metabolism. Since SREBPs are the key transcription factors in the pathogenesis of lipogenic gene expression, we investigated the effects of SREBP-1/2 in regulating lipid metabolism by targeting the expressions of FASN and HMG-CoAR. Our data demonstrated that the expressions of SREBP-1/2 were decreased in OH-induced steatosis HepG2 cells after MLE and nCGA treatment. These findings suggest that the suppression of FASN and HMG-CoAR in steatohepatitis is a crucial part of a protective positive regulatory feedback mechanism aimed at limiting the progression of glucolipotoxicity-induced steatohepatitis by preventing excessive triglyceride and cholesterol synthesis in hepatocytes.

The involvement of inflammation in steatohepatitis suggests that TNF-α/NF-κB/IL-6, as transcriptional regulators, can promote hepatic and adipose inflammatory responses and induce hepatocyte apoptosis and necrosis. Hepatocyte and adipose tissue NF-κB binding activities were directly associated with the magnitude of hepatic injury, and lowering NF-κB binding activity and TNF-α gene expression could attenuate the develop-ment of steatohepatitis. A recent study reported that suppressing the TNF-α/NF-κB sig-naling-mediated macrophage pathway could attenuate steatohepatitis in a mice model [36]. Park et al. also demonstrated that the inhibition of hepatic inflammation mediated by NF-κB could protect against steatohepatitis [37]. Other data suggest that IL-6 may be in-volved in inflammation and insulin resistance, while TNF-α may induce the progression of NAFLD and promote the development of steatohepatitis [38]. However, we also observed that treatment with miR-34a mimic or miR-34a inhibitor did not alter the profile of inflammation-related proteins (data not shown). MLE or nCGA may be affected by other microRNAs to modulate inflammatory pathways. Our results demonstrated that MLE and nCGA improved glucolipotoxicity-induced steatohepatitis and also con-tributed to the anti-inflammatory effects of the TNF-α/NF-κB/IL-6 signaling pathway in a cell model. We believe an interdisciplinary approach is needed for the management of pa-tients with type 2 diabetes and steatohepatitis, starting with early identification and stea-tohepatitis-specific therapy.

In our previous study, we showed that MLE could be used to ameliorate NAFLD through the molecular mechanisms of lipid synthesis and oxidation [20]. The polyphenolic compounds contained in MLE have been isolated and identified, and found to be rich in three CGA isomers, namely nCGA, Crp and CGA. In this study, we found that nCGA had the strongest alleviation effect on lipid accumulation when compared with the other two CGA isomers. This suggests that nCGA is the major functional component of MLE, which alleviates diabetic steatohepatitis by modulating the miR-34a/SIRT1/AMPK pathway.

Our results support that MLE and nCGA may protect against glucolipotoxici-ty-induced steatohepatitis through their inverse associations with lipogenesis and in-flammation. Several mechanisms were involved, including downregulation of miR-34a, leading to activation of the SIRT1/AMPK pathway and subsequently modulation of the expressions of lipogenesis and lipid β-oxidation enzymes as well as lipogenesis-associated transcription factors including SREBP-1, SREBP-2, FASN, and HMG-CoAR, and attenuation of inflammatory responses via the inhibition of NF-κB and TNF-α-related signaling.

## 4. Materials and Methods

### 4.1. Chemicals and Reagents

Dulbecco’s modified Eagle’s medium (DMEM), L-glutamine, penicillin -streptomycin mixed antibiotics, fetal bovine serum (FBS), trypsin-EDTA, and phosphate-buffered saline (PBS) were purchased from Gibco/BRL (Gaithersburg, MD, USA). Primary antibodies against SREBP-1, SREBP-2, HMG-CoAR (HMGCR; sc-271595), AMPK (sc-74461), CPTI (sc-20669), and PPARα (sc-9000) were obtained from Santa Cruz (Santa Cruz, CA, USA). Monoclonal antibodies against phosphor-AMPK (#2535), FASN (#3180) and α-tubulin were obtained from Cell Signaling Technology Inc (Beverly, MA, USA).

### 4.2. Preparation of MLE and CGA Isomers

MLE solution was prepared with ddH_2_O to a stock concentration of 50 mg/mL, and then filtered through a 0.22-μm filter for use in subsequent experiments. CGA, Crp and nCGA were dissolved in dimethylsulfoxide (DMSO) to prepare a stock solution with a concentration of 100 mM, and then diluted as required for subsequent experiments.

### 4.3. Cell Culture

Human hepatoma cell line HepG2 was grown in DMEM that contain 1 mM streptomycin/penicillin (Gibco BRL Co., Gaithersburg, MD, USA), 10% FBS (Gibco BRL Co., Gaithersburg, MD, USA), 2 mM L-glutamine (Gibco BRL Co., Gaithersburg, MD, USA), and 1 mM sodium pyruvate (Hyclone, GE Healthcare, Pittsburg, PA, USA) under-maintained at 37 °C, 95% humidity and 5% CO_2_, and the cells were obtained from the American Type Culture Collection (ATCC, Manassas, VA, USA).

According to previous studies, the condition of oleic acid (OA, 300 μM) was used to induce lipogenesis and fatty acid oxidation [39]. Therefore, in order to establish a glucolipotoxicity model, we co-treated OA (300 μM) and high glucose (25 mM) for subsequent experiments.

### 4.4. Cell Viability Assay

HepG2 cells (7 × 10^4^/per well) were seeded into 24-well plates for 24 h. MLE and the three CGA isomers (CGA, Crp, nCGA) were then added at different concentrations for 24 and 48 h. The cells were washed twice with PBS, and the culture medium was replaced with medium containing MTT (0.5 mg/mL) for 4 h. The culture medium was then removed, and 1 mL of isopropanol was added to dissolve the formazan crystals formed. Cell viability was analyzed using an ELISA reader with a wavelength of 563 nm.

### 4.5. Nile Red Analysis

Nile red is a lipophilic fluorescent dye capable of detecting intracellular lipids [40]. HepG2 cells were treated with OH medium to induce excessive lipid accumulation and simulate the development of diabetic steatohepatitis, and were treated with different concentrations of MLE and nCGA. After 24 h, the cells were detached with trypsin and washed 3 times with PBS. Nile red (1 µg/mL) was added for 30 min, after which the cells were washed with PBS and measured by flow cytometry. The intracellular lipid content was then analyzed using FACScan and CellQuestTM Pro software (Becton Dickinson Bioscience, San Jose, CA, USA).

### 4.6. miRNA Extraction and Real-Time PCR

Total RNA of HepG2 cells was collected using NucleoZOL reagent (Macherey-Nagel, Duren, Germany) according to the manufacturer’s protocol. The RNA was translated to cDNA, and quantitative real-time (qRT) PCR was then performed using a TaqMan^®^ MicroRNA Reverse Transcription Kit (Applied Biosystems, Carlsbad, CA, USA). In each sample, the PCR results were normalized to the endogenous control RNU6B. MiR-34a expression levels in the treated cells were compared to untreated controls using Light Cycler software.

### 4.7. Cell Transfection

HepG2 cells were seeded in a 6 cm dish, and after 24 h of incubation, the medium was replaced with antibiotic-free medium containing 10% FBS. The cells were transfected using a custom TOOLS Water DNA, RNA extraction kit (Biotools Co., Ltd., New Taipei, Taiwan) and RNA system according to the manufacturer’s protocol. The miR-34a inhibitor (5′-ACAACCAGCUAAGACACUGCCA-3′) and the miR-34a mimic (5′-UGGCAGUGUCUUAGCCUGG UUGU-3′) were added to serum-free medium and then mixed with T-Pro non-liposomal transfection solution for 15 min at room temperature. The mixture was added to each well of the 6 cm dish and incubated for 24 h. To verify the transfection efficiency, changes in miRNA expression were detected by qRT-PCR.

### 4.8. Western Immunoblotting

Total cell lysates were mixed with an equal volume of RIPA buffer, then heated for 10 min, analyzed using 8–12% sodium dodecyl sulfate polyacrylamide gel electrophoresis (SDS-PAGE), and transferred to a nitrocellulose membrane (Millipore, Bedford, MA, USA). Blots were reacted with slow shaking in blocking buffer (5% nonfat milk in PBS) for 1 h, probed with protein-specific primary antibodies at 4 °C overnight, washed with Tris Buffered Saline Buffer with Tween 20 (TBST), and then incubated with peroxidase-conjugated secondary antibodies for 1 h at room temperature. Finally, antigen-antibody complexes were developed using enhanced chemiluminescence (ECL) blotting detection reagents (Amersham Biosciences, Mountain View, CA, USA). Relative image density was analyzed using a Fuji LAS-3000 imaging system (FUJFILM, Tokyo, Japan).

### 4.9. Statistical Analysis

The experiments in each group were repeated three times, and the results were shown as mean ± standard deviation (mean ± SD). The results were plotted using GraphPad Prism 7 software (GraphPad Software, San Diego, CA, USA) and then analyzed with the Student’s *t*-test. One-way analysis of variance was used, followed by Duncan’s multiple range test to analyze differences between groups. Statistical analysis was performed using SPSS v22.0 (IBM Inc., Armonk, NY, USA). A *p* value < 0.05 was considered to indicate a significant difference.

## Data Availability

Not applicable.
